# Gender differences in peer reviewed grant applications, awards, and amounts: a systematic review and meta-analysis

**DOI:** 10.1186/s41073-023-00127-3

**Published:** 2023-05-03

**Authors:** Karen B. Schmaling, Stephen A. Gallo

**Affiliations:** 1 Department of Psychology, Washington State University, Vancouver, WA USA; 2 Scientific Peer Advisory and Review Services, American Institute of Biological Sciences, Herndon, VA USA

**Keywords:** Gender, Grant proposal, Grant award, Peer review

## Abstract

**Background:**

Differential participation and success in grant applications may contribute to women’s lesser representation in the sciences. This study’s objective was to conduct a systematic review and meta-analysis to address the question of gender differences in grant award acceptance rates and reapplication award acceptance rates (potential bias in peer review outcomes) and other grant outcomes.

**Methods:**

The review was registered on PROSPERO (CRD42021232153) and conducted in accordance with PRISMA 2020 standards. We searched Academic Search Complete, PubMed, and Web of Science for the timeframe 1 January 2005 to 31 December 2020, and forward and backward citations. Studies were included that reported data, by gender, on any of the following: grant applications or reapplications, awards, award amounts, award acceptance rates, or reapplication award acceptance rates. Studies that duplicated data reported in another study were excluded. Gender differences were investigated by meta-analyses and generalized linear mixed models. Doi plots and LFK indices were used to assess reporting bias.

**Results:**

The searches identified 199 records, of which 13 were eligible. An additional 42 sources from forward and backward searches were eligible, for a total of 55 sources with data on one or more outcomes. The data from these studies ranged from 1975 to 2020: 49 sources were published papers and six were funders’ reports (the latter were identified by forwards and backwards searches). Twenty-nine studies reported person-level data, 25 reported application-level data, and one study reported both: person-level data were used in analyses. Award acceptance rates were 1% higher for men, which was not significantly different from women (95% CI 3% more for men to 1% more for women, *k* = 36, *n* = 303,795 awards and 1,277,442 applications, *I*^2^ = 84%). Reapplication award acceptance rates were significantly higher for men (9%, 95% CI 18% to 1%, *k* = 7, *n* = 7319 applications and 3324 awards, *I*^2^ = 63%). Women received smaller award amounts (*g* = -2.28, 95% CI -4.92 to 0.36, *k* = 13, *n* = 212,935, *I*^2^ = 100%).

**Conclusions:**

The proportions of women that applied for grants, re-applied, accepted awards, and accepted awards after reapplication were less than the proportion of eligible women. However, the award acceptance rate was similar for women and men, implying no gender bias in this peer reviewed grant outcome. Women received smaller awards and fewer awards after re-applying, which may negatively affect continued scientific productivity. Greater transparency is needed to monitor and verify these data globally.

**Supplementary Information:**

The online version contains supplementary material available at 10.1186/s41073-023-00127-3.

## Background

Funding is needed to conduct most research and is key to scientists’ success. For example, extramural funding was among the criteria for tenure and promotion in the majority of a random sample of 92 biomedical sciences faculties, selected from among the top 852 universities in the world based on publication productivity [1]. Funded applications reflect positive evaluations of the significance and proposed methods of the research, and of the expertise of the investigator.

Around the world, women comprise an estimated 29.3% of researchers, which varies by country [2]. Women are underrepresented among researchers: in the United States (US), for example, women comprised 35.2% of doctorates in science, engineering, and health, but only 29.8% – a gap of 5.5% –was substantially engaged in research in 2017 [3]. Concerns about women’s underrepresentation in the sciences have focused on the leaky pipeline, unsupportive environments, and lack of mentoring, among other factors [4]. Biased evaluations, including peer reviewed grant applications, have been posited as another potential cause for differential success in the sciences [4]. Biases may be explicit or implicit, as reflected in overt discrimination or in automatic preferences, respectively. Men are more strongly associated with science than are women; this robust implicit bias effect has been replicated in 60 countries [5] and among STEM faculty at highly ranked universities in the US [6]. It seems plausible that biases and preferences may affect grant application and review processes, resulting in gender differences.

Evidence of gender bias in peer review has been inconsistent. Some narrative reviews have concluded that there is no evidence for gender bias in grant peer review [7, 8]. Other narrative reviews provide a mixed picture. For example, men have been observed to apply for grants more than women, but the proportions of successful applicants are similar [9]. Another review cited studies that showed both gender differences and lack of differences in grant applications outcomes [10], which are sensitive to context [11].

Quantitative reviews have been rare. Two studies used different analytic methods to examine a dataset comprised of 66 gender comparisons from 21 studies that spanned the years 1987–2005 [12, 13]. The earlier study compared odds ratios of applications and awards among women to those among men. They found that men were 7% more likely to receive an award than women, which was statistically significant, albeit associated with a small effect size [12]. The later study used more complex multilevel analyses that controlled for discipline, country, and year, and did not find significant gender differences [13].

Moderating variables, such as the nations studied, have contributed to an inconsistent literature on the association between gender and grant outcomes [11, 13]. Recent gender equity efforts in the EU include a mandate that “gender… (is) a ranking criterion for proposals with the same score” [14], which may result in better gender parity than in nations without such laws, including the United States (US).

The purpose of this study was to conduct a systematic review and meta-analysis of the literature since 2005 regarding gender differences in grant outcomes by conducting meta-analyses and mixed models of each outcome. This study addressed the question of gender differences in grant applications, awards, award acceptance rates, award amounts, and reapplication award acceptance rates. We also examined nation (US vs. non-US) as a potential moderator of gender differences [11, 13].

## Methods

The review was conducted in accordance with PRISMA 2020 standards [15].

### Protocol registration

The study protocol was submitted to PROSPERO on 19 January 2021 and was registered on 19 February 2021 (#CRD42021232153). This paper differs from the registered protocol as follows: the protocol did not list all of the measures of effect for each outcome measure; two of the proposed subgroup analyses (the type/level of grant mechanism and the gender of the study authors) could not be addressed in the scope of this paper.

### Search strategy and study identification

Three search engines were chosen to provide comprehensive coverage of the literature after consultation with an academic librarian: Academic Search Complete, PubMed, and Web of Science. The keywords used were “peer review” AND grant AND gender; the range of publication dates was 1 January 2005 to 31 December 2020; languages were English or French. The searches were last performed on 2 February 2021. The beginning date was chosen based on the most recent year in the previous quantitative reviews [12, 13].

Three people independently searched each database in January 2021 and compared the number of results. There was perfect agreement on the number of results from each search on each database. Next, two people independently screened the abstracts identified by each search and judged if each abstract clearly met or did not meet inclusion criteria.

The full text article of each possibly relevant manuscript was obtained and independently reviewed by two people to determine if it met inclusion criteria. The results of the three searches and the decision to include or exclude each study were combined and duplicate studies were eliminated. Relevant data (see below) were extracted by one person; all extracted data were checked by a second person. For articles in which incomplete data were reported, up to three emails were sent to corresponding authors to request additional data.

Additional relevant articles were found based on forward and backward searches of the articles identified through the systematic review. The reference lists of articles that met inclusion criteria were reviewed for earlier possibly relevant articles. Each search engine was used to identify later possibly relevant publications that had cited articles that met inclusion criteria.

### Inclusion and exclusion criteria

To be included, the study needed to report data on numbers of peer reviewed grant applications, awards, or reapplications, or amounts of funding (mean or median and standard deviation or interquartile range) separately by gender. Studies were excluded that reported none of these variables, if the data had been reported in the previous review [12, 13], or if the data in the study were superseded by a more comprehensive report. The last inclusion criterion was intended to identify independent, nonduplicated data sources: for example, a report of R-type applications by men and women otolaryngologists to the NIH in 2015 would be superseded by data from all NIH institutes regarding R-type applications from 2005–2020. If a report contained both unique data not included in another report and data superseded by another report, only the unique data were retained.

### Extracted variables

Characteristics of the included studies were extracted including the country, type or name of the granting agencies, years studied, data source (data extracted from archives or obtained through survey), characteristics of the participants, if participants were referred to by sex (female, male) or gender (women, men) based on biological or socially-defined characteristics, respectively, grant type or mechanism, data on eligible populations (as included in the study or obtained through searching for data that reflected study characteristics including country, year, disciplines, and sector, e.g., higher education), and presence or absence of each outcome variable and the level of data reporting (person-level, application-level, or both). Finally, each study’s assessment of gender bias was extracted, noting if there was a conclusion that there was bias (or similar phrases including difference, gap, disparity, discrepancy, was inequitable, etc.), was no bias, the results were mixed or equivocal, or did not make this determination (i.e., only reported data), accompanied by a quotation from the article supporting the assessment.

Extracted outcome variables, by gender, were the number of grant applications, number of awards, award acceptance rate (awards divided by applications), amounts of awards, number of reapplications, number of awards after reapplication, and reapplication award acceptance rate. Proportions of applications, awards, reapplications, and awards after reapplications were calculated by the number for women divided by the total for men and women. For studies that reported more than one data source, data from each was extracted and preserved on a separate row. Reapplications and resubmissions were of two types: competitive renewals (e.g., a competitive renewal application following an R01 award) or resubmissions following unfunded applications. Award amounts were standardized by first converting the original currency to 2021 values using on-line calculators (see the Supplementary file eMethods) and then converting to US$ using Google’s on-line currency converter. For studies that reported award amounts’ medians and interquartile ranges, a macro estimated means and standard deviations (see the Supplementary file eMethods). Pre-specified moderator variables were extracted: the country in which the study was conducted (US or non-US).

The authors extracted and checked all data; disagreements were resolved by discussion.

### Data analysis

Datasets and analysis syntax are available on the project website. Descriptive statistics were calculated for each outcome variable, by gender. MetaXL version 5.3 [16] was used for the meta-analyses of gender differences using the raw (unweighted) data and compared subgroups of US versus non-US studies when 10 or more studies were available. Inverse variance heterogeneity models were used because of methodological diversity [17]. Forest plots were used to depict the effect size and 95% confidence interval (CI) of each study, grouped by US and non-US studies. Separate meta-analyses of gender differences were calculated for each outcome variable using the following effect sizes: award acceptance rates and reapplication award acceptance rates used rate differences; award amounts used Hedges’ *g*; and analyses of proportions (applications, awards, reapplications) used double arcsine transformed prevalence [18]. Sensitivity analyses were performed by excluding each study in turn [19] (see the Supplementary file eResults).

The dispersion of effect sizes was quantified with *Q* and *I*^2^ statistics. Doi plots and the LFK index were used to assess reporting bias [20]. Doi plots graph the effect on the x axis and the *Z*-score of the quantile on the y axis; a vertical line appears at the *Z*-score closest to zero. Heterogeneity was evident when the two limbs of studies’ effects on either side of the vertical line formed a non-symmetrical inverted V shape. The LFK index was the difference in the area under each limb of the plot on either side of the vertical line. Values of the LKF index less than -1.0 and greater than 1.0 reflected asymmetry in the distribution of the studies’ *Z*-scores compared to the effect (proportion, rate difference, or *g*).

In meta-analyses of proportions, forest plots and visual representations of effect size dispersion have been recommended to see the effects in individual studies but generalized linear mixed models with logit link function are recommended to estimate overall effects [21]. Thus, generalized linear mixed models (GLMM: IBM SPSS v28, Chicago IL) were used to estimate overall effects of gender and nation (US versus non-US) for the proportions of grant applications and awards, with the following assumptions: binomial probability distribution, logit link function, random effects for studies and intercepts; fixed effects for gender and nation (coded as US or non-US); repeated measure for gender. There were insufficient data to compare nations for the GLMMs of the proportions of application resubmissions or awards after resubmissions.

## Results

### Included studies

**Fig. 1:**
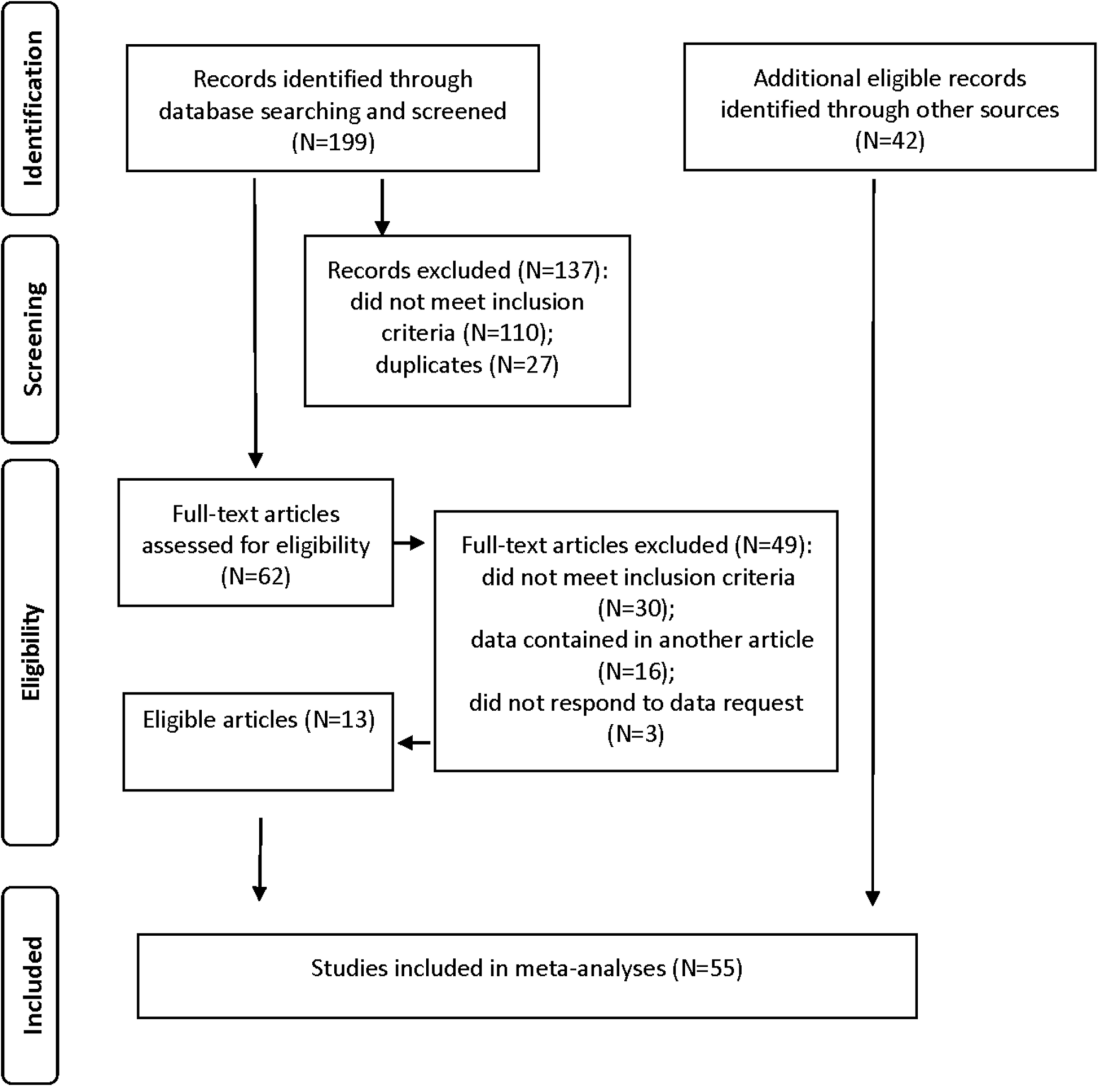
Flow Diagram

A flow diagram [22] is shown in Fig. 1. In summary, 241 studies were identified by the searches and other sources. After excluding studies that did not meet inclusion criteria, were duplicates, or contained data superseded by other studies, 55 studies provided data on one or more of the outcome variables [23–77].

These 55 studies provided data from 14 nations and the EU, reported data from 1975 to 2020 (see the Supplementary file eResults) from diverse funding sources: 45 funding sources were named in the studies; additionally, five studies examined multiple funding sources (e.g., government, industry, foundation, intramural) but did not list the specific funders [34, 40, 42, 72, 73]. Most studies reported complete archival data on their population except for four studies that surveyed samples of participants [23, 40, 58, 72]. Fourteen studies only referred to investigators by gender (25%), one study only referred to investigators by sex (2%, “male”, “female”), and 40 studies (73%) used both sex- and gender-linked terms regarding investigators.

### Award acceptance rate

There were 29 sources comprising 36 samples that provided data for this meta-analysis (*n* = 303,795 awards and 1,277,442 applications). (See Table 1 for meta-analysis results; see the Supplementary file eResults for forest plots, Doi plots and sensitivity analyses.) Unweighted average award acceptance rates were 22.7% for women and 24.3% for men. The pooled effect revealed a 1% lower award acceptance rate for women than men (95% CI 3% more for men to 1% more for women), which was not significantly different. Individual studies ranged from a 17% greater award acceptance rate for men in an intramural program [41] to a 5% greater award acceptance rate for women in a grant-writing program [74]. There was significant heterogeneity (*Q* = 212.96, *p* < 0.001, *I*^2^ = 84%) with evidence of reporting bias (LFK = -2.85). Among these 36 samples, the pooled award acceptance rate was 2% higher for men than women in non-US nations and 1% higher for men than women in the US, which was not significantly different.

**Table 1:** Meta-analysis results

				Subgroup Comparison Effect Sizes (95% CI)	
	Pooled Effect Size (95% CI)	*Q*^a^, *I*^2^	LFK Index^b^	US	Non-US
Award acceptance rates	-1% (-3,1)	213, 84%	-2.85	-1% (-3,2)	-2% (-3,-1)
Reapplication award acceptance rates	-9% (-18,-1)	16, 63%	-0.44	N/A	N/A
Award amounts	-2.28 (-4.92, 0.36)	119,417, 100%	0.67	-5.11 (-11.63, 1.41)	-.24 (-.31,-.17)
Proportions of applications	30% (22,38)	17,060, 100%	2.87	30% (19,40)	31% (26,36)
Proportions of reapplications	31% (24,37)	195, 95%	3.37	N/A	N/A
Proportions of awards	24% (14,34)	15,711, 100%	7.07	23% (11,36)	29% (24,34)
Proportions of awards after reapplication	30% (19,42)	117, 92%	2.82	N/A	N/A

^a^All *Q* statistics were significant *p* < .01; decimals were omitted for space considerations. ^b^LKF indices < -1.0 or > 1.0 reflect reporting bias. Note: Negative award acceptance rates and amounts reflect higher values for men than women

### Reapplication award acceptance rate

There were seven samples from six sources in this meta-analysis (*n* = 3,324 awards and 7,318 applications). The pooled funding rate after reapplication was significantly (9%) lower for women than men (*k* = 7; 95% CI 18% to 1%): unweighted data revealed a 38% funding rate for women versus 48% for men with moderate heterogeneity (*Q* = 16.28, *p* = 0.01, *I*^2^ = 63%) and no significant reporting bias (LFK = -0.44). Individual studies ranged from no gender difference among early career investigators in the Netherlands [71] to a 42% higher funding rate for men in otolaryngology who had previously received small grants [33]. There were five samples from the US and two from outside of the US, which were insufficient for nation comparisons.

### Award amounts

There were 13 samples from nine sources that provided data for this meta-analysis (*n* = 212,935 awards). The unweighted averages (and standard deviations) were $341,735.54 ($275,465.79) for women and $659,081.00 ($967,813.34) for men. Men received significantly larger award amounts than did women by a factor of 2.28 (95% CI 4.92 to 0.36). While all studies reported larger awards to men, the gender differential ranged from 0.11 for a Canadian program in the cognitive sciences [69] to a factor of 5.13 for NIH research project grants [52]. There was significant heterogeneity (*Q* = 119,416.86, *p* < 0.001, *I*^2^ = 100%), but no significant reporting bias (LFK = 0.67). In subgroup comparisons, the disparity between men’s and women’s award amounts was greater in the US than in non-US nations (factor of 5.11 compared to 0.24).

### Proportions of applications

Thirty-two sources provided 39 independent samples of applications (*n* = 1,311,5552, median per source = 3615, IQR = 15,464). Women accounted for 30% of applications (95% CI 22% to 38%). As shown in the forest plot, the proportion of women applicants ranged from 13% in the agricultural sciences [51] to 70% among a small cohort of biomedical researchers in a grant writing coaching program [74]. There was significant heterogeneity in effect sizes (*Q* = 17,060, *p* < 0.001, *I*^2^ = 100%) and in data sources with numbers of applications ranging from 64 to over 771,000. There was significant reporting bias (LFK = 2.87).

In subgroup comparisons of these 39 samples, there were similar pooled proportions of applications from women in US and non-US studies (30% and 31%, respectively). The results of the GLMM procedure showed a strong main effect for gender (*F*(1,74) = 649.80, *p* < 0.001, but the main effect of nation and the gender by nation interaction were not significant (*F*(1,74) = 0.00 and 1.08, *p* = 1.00 and 0.30, respectively).

### Proportions of reapplications

Application resubmissions were reported by ten sources (*n* = 44,138, median per source = 899, IQR = 3526) among previously successful (i.e., awardees) and unsuccessful applicants: women accounted for 31% of resubmissions (95% CI 24% to 37%). In individual studies the proportions of women reapplicants ranged from 23% in a study of Harvard Medical School faculty [73] to 62% among obstetrics-gynecology K awardees [54]. There was significant heterogeneity (*Q* = 195.04, *p* < 0.001, *I*^2^ = 95%) and evidence favoring reports of greater proportions of applications from women (LFK = 3.37). The results of the GLMM procedure revealed a strong main effect for gender (*F*(1,18) = 534.54, *p* < 0.001). There were insufficient studies to conduct nation comparisons.

### Proportions of awards

Forty-one sources provided 47 independent samples of the proportion of awards by gender (*n* = 615,653 awards, median per source = 2377, IQR = 3785): women accounted for 24% of awards (95% CI 14% to 34%). The proportion of awards to women ranged from 17% in a Swiss program [37] to 72% among grant-writing program participants [72]. Reflecting the diversity in methodology, there was significant heterogeneity (*Q* = 15,711.24, *p* < 0.001, *I*^2^ = 100%) and evidence favoring reports of greater proportions of awards to women (LKF = 7.07).

Using these 47 samples, the proportion of awards to women was 6% higher in non-US nations than in the US (29% and 23%, respectively). The results of the GLMM procedure showed a strong main effect for gender (*F*(1,88) = 437.08, *p* < 0.001, and a gender by nation interaction (*F*(1,88) = 11.11, *p* = 0.001, but the main effect of nation was not significant (*F*(1,88) = 0.00, *p* = 1.00). The proportion of awards to women in the US was smaller than to women elsewhere, and the proportion of awards to men in the US was larger than to men elsewhere.

### Proportions of awards after reapplications

Based on ten sources, women received 30% of awards (*n* = 156,574, median per source = 221, IQR = 854) after reapplication among previously funded scientists (95% CI 19% to 42%). The proportions of reapplying women awardees ranging from 8% among otolaryngologists who had previously received small grants [31], to 60% among previous obstetrics-gynecology K awardees [54] and among previous developmental psychopathology T recipients [60]. There was significant heterogeneity (*Q* = 116.83, *p* < 0.001, *I*^2^ = 92%) and evidence favoring reports of greater proportions of awards to women (LFK = 2.82). The results of the GLMM procedure showed a strong main effect for gender (*F*(1,18) = 3487.11, *p* < 0.001). There were insufficient studies for nation comparisons.

### Sensitivity analyses

Sensitivity analyses showed the extent to which excluding some of the large datasets would alter the outcomes, especially the NIH data [52]. Regarding the award acceptance rate and amount comparisons, when each study was excluded in turn, excluding the NIH data would have increased men’s greater award acceptance rate to 1.6% from a pooled rate of 1.0%, and men’s greater award amounts would have decreased to a factor of 0.24 more than women’s from a pooled factor of 2.28. For the proportion of applications, excluding the NIH data would decrease the proportion of applications from women to 28.5% from a pooled proportion of 30% with all studies included. For the proportion of awards, excluding the NIH data would have decreased the proportion of awards to women to 21.6%, compared to 24% with all studies included. Excluding the NIH data would have decreased the proportion of women receiving awards after resubmission to 27.3% from 30%. Finally, the sensitivity analyses of the proportions of application resubmissions and reapplication award acceptance rates showed small changes, less than 1%, when studies were excluded. None of the sensitivity analyses substantially changed heterogeneity such that statistically significant *Q* and large *I*^2^ statistics were no longer so.

### Representation of eligible researchers by gender

It is important to place these results in the context of the proportion of eligible researchers by gender. Supplementary file eResults (eTable2) summarizes the proportion outcomes for each study (from eFigures 10, 13, 16, 19).

Proportions of eligible women researchers were estimated for each study (*n* = 13,553,340, median per source = 17,585, IQR = 119,387). The pooled estimate of eligible women was 36% (95% CI 33% to 39%). As shown in the forest plot, the proportion of eligible women ranged from 13% in Italian agricultural sciences [51] to 67% among pediatric residents [41], developmental psychobiology postdoctoral trainees [60], and biomedical researchers in a grant writing coaching program [74]. There was significant heterogeneity in effect sizes (*Q* = 130,375, *p* < 0.001, *I*^2^ = 100%) and significant reporting bias (LFK = 2.07).

For each study, two columns in eTable 2 appraise the application/reapplication and award/award after reapplication proportions as less than, greater than, or equivalent to the eligible proportions (see Supplemental file eMethods). Fewer women applied than were eligible in 54% of studies (25 of 46); more applied than were eligible in 22% of studies (10 of 46); and the proportions were equivalent in 24% of studies (11 of 46). Fewer women received awards in 65% of comparisons to eligible proportion estimates (33 of 51); more women in 20% (10 of 51); and equivalent proportions in 16% (8 of 51) (percentages are rounded). In the majority of papers, women were less likely to apply and to receive awards compared to those who were eligible.

## Discussion

A systematic review of the 2005–2020 literature yielded 55 sources of gender data on peer reviewed grants, predominantly from Europe and North America. The proportions of women that applied for grants (30%), re-applied (31%), accepted awards (24%), and accepted awards after reapplication (30%) were less than the proportion of eligible women (36%). However, the award acceptance rate was similar for women and men, implying no gender bias in this peer reviewed grant outcome. Additionally, women received smaller award amounts and fewer awards after re-applying, but these estimates were based on smaller numbers of studies.

This lack of gender difference in award acceptance rate is consistent with earlier observations [9, 13, 56, 78]. A previous analysis of 1987–2005 grant award acceptance rates from Australia, western Europe and the United States found a 7% higher award acceptance rate for men albeit with a small effect size [12], but multilevel analyses of the same data that controlled for several factors found insignificant gender effects [13]. The 7% disparity was greater than the 1% higher award acceptance rate for men found in this study, which was not significant because the 95% confidence interval included zero. Thus, both current and past [13] reviews found nonsignificant gender differences for award acceptance rates. The previous review focused on one outcome – the gender difference in award acceptance rates – and all of the studies in their review provided data on this outcome. The present study examined other outcomes in addition to award acceptance rate and the outcomes reported by each source varied: not all sources provided data on all outcome variables. Ideally, all outcome variables would be available from each source to facilitate interpretation and comparison. The previous review performed a broader search (seven databases compared with three in the present study), but both studies used similar search terms (peer review; gender) and the sources were from similar geographic regions. The previous review used multilevel models; our study used traditional meta-analyses to examine gender albeit with a novel inverse variance heterogeneity model [17], augmented by multilevel models for outcomes expressed as proportions.

To further compare our data with the previous review [12, 13], we input their data, aggregated all data for each source and conducted analyses of the proportions of applications and awards as we had done in the present study. The pooled prevalence of the proportions of women’s applications and awards were 21% and 19% based on the earlier data, as compared to 30% and 24% in the present study, suggesting that there have been significant gains in the representation of women among applicants and awardees since 2005 (see eDiscussion), consistent with a recent narrative review [11].

Persistence and securing continued funding are necessary for continued scientific productivity and advancement. Women submitted 31% of reapplications, however, their reapplication acceptance rate was significantly lower (9%) than men. The gap between the pooled gender disparities for the general award acceptance rate and the reapplication acceptance rate – 1% versus 9% – may result from underlying variability in the studies that contributed data to each statistic. Some of the reapplication studies examined reapplication of the same applications, and some examined continued funding or advancement in research, such as awards of research grants among previous career or small grant awardees.

Peer review of grant applications typically includes an evaluation of the investigator’s suitability to conduct the research, based on their past accomplishments, such as prior publications. Evaluations of women’s research accomplishments suffer when their research is devalued and their publications are less cited [79]. Increasingly, however, the use of metrics such as journal impact factor is discouraged, e.g., by projects DORA/TARA [80]. Women are less represented as academic rank increases, although the causes of women’s lesser representation are unclear [2]. Relatively few studies reported data on resubmissions after unsuccessful applications, competitive renewals, or maturation in funding mechanisms. In general, women resubmitted applications in similar proportions to overall applications: about 31%. Women comprised proportionally more of those with awards after resubmissions (30%) than women with general awards (24%).

Women’s 9% lower award acceptance rate after reapplication may reflect possible bias and the leaky pipeline [4] more than any other result in this study. This result was also consistent with the majority of studies’ conclusions that there was gender bias favoring men (see eResults). Male applicants have reported receiving more constructive feedback from peer reviewers than did female applicants [81], which may contribute to better outcomes after reapplication. Women were more likely to interpret peer reviewers’ feedback more negatively than did men, which in turn was related to less intention to reapply [28]. Women may be more discouraged by grant rejections than men, contributing to differential research productivity and longevity [82]. Women’s lesser success than men’s after reapplying for grants sheds no light on the potential processes contributing to this phenomenon, which are worthy of study. Furthermore, a focus on outcomes, as in the present study, does not address the complex interaction of individual, systemic, and social barriers that may produce and maintain such outcomes [11, 79].

Women received significantly smaller award amounts than did men. This effect was especially pronounced for US-based studies: nation has been suggested as a potential moderator of gender differences in previous studies [10–13]. While the comparison of US and other nations’ amounts is novel, others have observed that women request and receive smaller award amounts than men. This effect could be because of different types and costs of research, lower salaries [83], or differential entitlement resulting from professional mentoring or other qualities [45]. Administrative budget reductions also could be a source of gender disparities. NIH awards are often less than requested: for example, the National Institute of Aging reduced continuing awards by an average of 20% in FY 2022 [84]. However, it would be unusual for other funders to award a lesser amount than requested, such as the Wellcome Trust [85]. In two papers in our review, women and men received similar proportions of their requested amounts in one [73], and in the other, men’s awarded amounts were significantly greater than women’s although their requested amounts did not differ [56], which the authors posit could be due to a bias against women engaging in risky research. Investigator requests, peer reviewer recommendations, and administrative decisions are all potential sources of gender differences in grant award amounts.

In comparisons of the US to non-US studies, there was a small difference in the proportion of applications submitted by women (1% more in non-US studies) and a 6% higher proportion of awards to women in non-US studies. These findings are potentially consistent with a 2020 report from the World Economic Forum in which the US ranked 53^rd^ in gender equality among 153 countries [86]. In contrast to the US, gender equity laws and policies exist in many EU countries [14].

It is important to place these results in the context of the representation of researchers by gender but challenging to do so. In the majority of studies, women were less likely to apply and to receive awards compared to estimates of eligible populations. However, these comparisons should be interpreted cautiously. Some estimates of the eligible population were precise (e.g., [63]), but other estimates were less specific to the sample. For example, some studies’ eligible proportions were based on all researchers whereas the grant applications were for early career researchers (e.g., [48]) and unsurprisingly, women’s share of applications was greater than the eligible proportion because women are better represented in the earlier academic ranks.

This study contributes to the reviews of gender participation in peer reviewed grants and is the only review of which we are aware to use systematic review and meta-analytic techniques since the 2007 and 2009 papers [12, 13]. It is also unique in its consideration of several variables reflecting different aspects of participation and review. It also had several limitations. First, the data used in the study did not include all nations, mechanisms, and the entire 2005–2020 period. The sources were limited to those identified by searching the published literature and those cited by or citing the published literature, which included some reports from nations’ funders. It would be valuable to conduct a systematic review based on funders’ reports. Population data from funding sources would likely provide more stable estimates of outcomes. A search of the Pivot-RP database of funders (ProQuest, Inc) identified 8,857 government (national, state, local), industry, foundation, institutional, private, and other funders in the geographic regions of the studies in our review. Thus, our report stemming from the published literature represents data from a minority of possible funders. As shown in Fig. 1, records in the meta-analysis found by the database searches were fewer than the additional sources identified by reviewing citated or citing articles of the records found through database searches. Some of the articles found through database searching were superseded by other sources or were included in the previous review (see Fig. 1). The additional sources were not identified by the database searches for several reasons. Of the 42 additional sources, 30 were indexed in PubMed; of those, 1 was not indexed, 7 (23%) were not indexed as gender; 10 (33%) were not indexed as grants or research support; and 15 (50%) were not indexed as peer review. The diversity of the additional sources suggests that the original search strategy was appropriate, but this literature had inconsistent index terms.

Some authors and funding agencies were responsive to our requests for additional data, but some did not respond or indicated they would not or could not provide data. For example, one national funding agency compiled data in response to our request, but its release to us was embargoed by its authorities. Such missing data could have contributed to the evidence of reporting bias found for most of the variables we examined.

Second, our goal was to use non-overlapping datasets. Most of the otherwise eligible studies identified by our searches were excluded because their data were superseded by another source. However, some overlap was still possible. For example, applications by Harvard school of medicine faculty to diverse funders probably included the NIH [73], but we were unable to examine applications separately by funding entity.

Third, we combined years of data because some sources could not be disaggregated by year or funding mechanism. The inability to disaggregate data by year and add a time variable to the analyses was a limitation of the study. The prior meta-analysis found no effect for the year that the study was published but noted that the year of the data collection may be a more appropriate comparator [12]. In future studies it would be valuable to examine the trajectories of change in award acceptance rates and other outcomes over time, particularly given the increased emphasis on gender equity initiatives and policies in recent years, some appear to result in a narrowing gender gap [2].

Sources varied in reporting data aggregated across funding mechanisms, or separately for different mechanisms. Although formal comparisons of outcomes for different mechanisms were beyond the scope of this paper, for example, women appeared to be more successful with personnel mechanisms (e.g., NWO “talent”, Swiss NSF career, NIH K) than research mechanisms (e.g., NWO “free competition”, Swiss NSF project, NIH research project grants). There would be value in examining data disaggregated by funding mechanism in addition to year.

Fourth, for some studies’ award amounts, we estimated the mean and standard deviation from median and IQR. Those studies’ data may not have been normally distributed, so the estimation of the mean and standard deviation may not be valid. Fifth, as most studies reported data on their population of interest, we did not conduct quality assessments on the small numbers of studies that surveyed samples. Sixth, studies were heterogeneous in size and focus, which may have contributed to the variability in effect sizes observed in most of the analyses.

Narrative reviews have stated that “grant peer review is a gender fair process” ([7], p. 3160] based on grant award acceptance rates. But conclusions about processes should not be inferred from outcomes [11]. Inferences about peer review processes should be based on process studies – for example, entailing blind reviews or experimental manipulation of gender pronouns – rather than on outcomes. While outcomes are suggestive, they are an insufficient basis for conclusions about processes. Also, a focus on variables beyond award acceptance rates is important to provide a more complete picture of gender similarities and differences. Differences in the submission of grant applications or the receipt of grant awards reflect different processes and involve different individuals, including investigators, mentors, peer reviewers, and scientific review officers. Additionally, the extant literature may include incorrect or misinterpreted information. For example, a widely cited study [45] reported that same number of applications by men to both NSF and NIH, and one of its authors affirmed that the number of applications to NSF was an error. This error raises concerns about others’ conclusions based on the data therein.

Future research on this topic would address the limitations in this study. Ideally, data would be disaggregated by year to inspect trends over time, and by gender or sex, with the latter concepts clearly identified and defined. Every source should provide person-level data on all outcome variables to facilitate the following comparisons: applicants to eligible applicants; rates of accepted awards to applications; reapplicants to eligible reapplicants; rates of accepted awards after reapplication to reapplications; awarded amounts to requested amounts. Moderator variables should include investigator variables of discipline, age, career level, institution type, percent of effort dedicated to research, previous productivity (papers, grants); funder variables of type (public, private), funding mechanism, and if the funder is subject to gender equity laws or policies; and contextual variables of author gender (e.g., proportion of women authors of the report) and proportion of eligible women or women in the discipline. The foundation of this study was a systematic review of the published literature, followed by forwards and backwards searches, which yielded heterogenous sources from small intramural grant programs to large, national funders. Analyses of more homogeneous sources would be valuable, such as data from national funders separately from private foundations, and from intramural mechanisms.

Evidence of differential gender participation in peer reviewed grant applications and awards has led to recommended [4] and implemented policies, such as “structural priority funding” to women ( [25], p. 3). Increasing the proportions of women grant peer reviewers is also recommended. A recent study of NIH study sections found women comprised about one-third of reviewers [87]. They recommended increasing women’s involvement in the review process for “opportunities to impact the nation’s research agenda” ([87], p. 3). More fundamentally, participation in peer review provides invaluable insight into the peer review process, to cutting edge ideas, and to new colleagues for collaboration, mentoring, and sources of external review letters. Europe has been a leader in policies to promote gender equality in research funding, such as Horizon Europe [14], gender quotas on review panels [88], and the Marie Sklodowska-Curie actions: the latter reported that from 2014 to 2018, women received 53.2% of the projects for experienced researchers [89]. Other countries’ gender equity policies and initiatives have not yet met with this level of success. In the UK, the representation of women professors in UK institutions of higher education grew from 24.2% in 2012/2013 to 26.8% in 2016/2017, but the growth was not clearly linked to institutions’ level of engagement with the Athena Scientific Women’s Academic Network (SWAN) initiative [90]. In the US, women faculty in STEM among awardees of NSF Institutional Transformation grants grew 8% to 24% and comparator institutions grew relatively less (5%) but had better overall representation (27%) [91]. Individuals’ outcomes – the focus of this review – cannot reflect cumulative systemic and social inequalities and disadvantages [11, 79] that are contextually important to and drivers of such outcomes. Equity initiatives hold promise to compensate for some sources of gender disparities.

## Conclusion

Women submitted only 30% of grant applications – less than those eligible to do so – but their award acceptance rate was similar to men. However, women received smaller award amounts and fewer awards after reapplying, which may negatively affect continued scientific productivity. These estimates were based on smaller numbers of data points than for award acceptance rates. Greater transparency in grant funding is needed to monitor and verify these data globally, and to allow for analysis of changes over time.

## Supplementary Information

**Additional file 1.**

## Data Availability

The analysis syntax and datasets supporting the conclusions of this article are available at the Open Science repository, https://osf.io/e5t4v/.
