# Supplementary material for: Gender differences in peer reviewed grant applications, awards, and amounts: a systematic review and meta-analysis
Source: Res Integr Peer Rev. 2023 May 3;8:2. doi: 10.1186/s41073-023-00127-3 (PMC10155348; doi:10.1186/s41073-023-00127-3)
Supplement: Supplementary file 1 — Additional file 1. [file 41073_2023_127_MOESM1_ESM.docx]

**Online Supplement**

Schmaling, KB, Gallo, SA. Gender differences in peer reviewed grant applications, awards, and amounts: A systematic review and meta-analysis.

**Table of Contents**

**eMethods** …………………………………………………………………………………….….. 3

Currency conversion calculators ……………………………………………………………….... 3

Mean and standard deviation calculator from median and IQR ……………………………….... 3

Rationale for +/- 3% margin to reflect differences between observed and eligible proportions ... 3

**eResults** …………………………………………………………………………………….…… 4

Studies’ conclusions about gender bias …………………………………………………………. 4

eTable 1. Characteristics of Included Studies ………………………………………………..….. 5

eTable 2. Included Studies’ Comparisons to Eligible Proportions of Women..………………... 12

eFigure 1. Gender Differences in Award Acceptance Rates in U.S. versus non-U.S. Studies: Forest Plot ……………………………………………………………………………………… 20

eFigure 2. Gender Differences in Award Acceptance Rates: Doi Plot ……………………….... 21

eFigure 3. Gender Differences in Award Acceptance Rates: Sensitivity Analyses …………… 22

eFigure 4. Gender Differences in Reapplication Award Acceptance Rates: Forest Plot……..... 23

eFigure 5. Gender Differences in Reapplication Award Acceptance Rates: Doi Plot ……..….. 24

eFigure 6. Gender Differences in Reapplication Award Acceptance Rates: Sensitivity Analysis …………………………………………………………………………………….…….…….… 25

eFigure 7. Gender Differences in Award Amounts in U.S. versus non-U.S. Studies: Forest Plot

………… ……………………………………………………………………………….…….… 26

eFigure 8. Gender Differences in Award Amounts: Doi Plot ……………………….……….… 27

eFigure 9. Gender Differences in Award Amounts: Sensitivity Analysis ……………..………. 28

eFigure 10. Gender Differences in the Proportions of Applications in U.S. versus non-U.S. Studies: Forest Plot ………………………………………………………………………….…. 29

eFigure 11. Gender Differences in the Proportions of Applications: Doi Plot ………..…….…. 30

eFigure 12. Gender Differences in the Proportions of Applications: Sensitivity Analysis ..…... 31

eFigure 13. Gender Differences in the Proportions of Application Resubmissions: Forest Plot …………...…………………………………………………………………………………….... 32

eFigure 14. Gender Differences in the Proportions of Application Resubmissions: Doi Plot .... 33

eFigure 15. Gender Differences in the Proportions of Application Resubmissions: Sensitivity Analysis ……………………………………………………………………………………….... 34

eFigure 16. Gender Differences in the Proportions of Awards in U.S. versus non-U.S. Studies: Forest Plot …………………………………………………………………………………….... 35

eFigure 17. Gender Differences in the Proportions of Awards: Doi Plot ……………….……... 36

eFigure 18. Gender Differences in the Proportions of Awards: Sensitivity Analysis …….….... 37

eFigure 19. Gender Differences in Proportions of Awards after Resubmission: Forest Plot .…. 38

eFigure 20. Gender Differences in Proportions of Awards after Resubmission: Doi Plot …….. 39

eFigure 21. Gender Differences in Proportions of Awards after Resubmission: Sensitivity Analysis ……………………………………………………………………………………….... 40

eFigure 22. Proportion of Eligible Women Applicants: Forest Plot …………………………... 41

eFigure 23. Proportion of Eligible Women Applicants: Doi Plot …………………………….... 42

eFigure 24. Proportion of Eligible Women Applicants: Sensitivity Analysis ……………….… 43

**eDiscussion** …………………………………………………………………………………..… 44

Search to estimate the number of funders …………………………………………………….... 44

Re-analysis of Bornmann et al., 2007 data …………………………………………………….. 44

eFigure 22. Gender Differences in Applications in Bornmann et al. (2007) data: Forest Plot .... 46

eFigure 23. Gender Differences in Awards in Bornmann et al. (2007) data: Forest Plot …….... 47

**eMethods**

**Currency conversion calculators**

Award amounts were standardized by first converting the original currency to 2021 values using on-line calculators at [www.in2013dollars.com](http://www.in2013dollars.com); [www.officialdata.org](http://www.officialdata.org); [www.worlddata.info/asia/hong-kong/inflation-rates.php](http://www.worlddata.info/asia/hong-kong/inflation-rates.php)) and then converting to US$ using Google’s on-line currency converter.

**Mean and standard deviation calculator from median and IQR**

For studies that reported award amounts’ medians and interquartile ranges, a macro estimated means and standard deviations (<https://bmcmedresmethodol.biomedcentral.com/articles/10.1186/1471-2288-14-135#citeas>).

**Rationale for +/- 3% margin to reflect differences between observed and eligible proportions**

All but one study provided one or more proportion outcomes. These observed proportion outcomes for women were compared to the estimated proportions of eligible women researchers. The observed proportion was considered less than or greater than the eligible proportion when the difference was < 3% or > 3%, respectively. The rationale for the 3% margin was based on a comparison of 30% (pooled proportion of applications from women) to 33% with sample sizes of 3615 (the median sample size of the applications), which yields p = .006 (MedCalc Software Ltd. Version 20.211; accessed January 9, 2023). Similarly, for proportions of applications (median sample size = 2377), pooled proportion of awards from women = 24% compared to 27%, p = .018). When the observed and eligible proportions differed by less than 3%, they were deemed equivalent.

**eResults**

**Studies’ conclusions about gender bias**

As shown in the eTable 2, 46 of the 55 studies in the review offered statements about the presence of gender bias and 9 studies did not. Among the 46 papers with conclusions, 24 (52%) concluded there was gender bias favoring men, 15 (33%) concluded there was no gender bias, and 7 (15%) reported mixed results or were equivocal.

eTable 1. Characteristics of Included Studies

| **1^st^ author, year [ref #]** | **Region** | **Agency type or name** | **Years** | **Data type** | **Level of Data** | **PIs** | **Mechanism** | **Apps^a^** | **Awds** | **Awd Rate** | **Amt** | **App & Awd Persist** |
| --- | --- | --- | --- | --- | --- | --- | --- | --- | --- | --- | --- | --- |
| Akabas, 2019 [23] | US | NIH | 1975-2014 | Survey | Person | MD-PhDs | Research grant |  | X |  |  |  |
| Andersson, 2021 [24] | Sweden | Karolinska  Institutet | 2014-17 | Archive | Person | All eligible | Personnel | X | X | X |  |  |
| Australian Gov, 2019-20 [25] | Australia | NHMRC | 2019-20 | Archive | Application | All eligible | Personnel | X | X | X |  |  |
| Bautista-Puig, 2019 [26] | Europe | ERC | 2007-16 | Archive | Application | All eligible | Start., adv., consolidator | X | X | X |  |  |
| Beck, 2017 [27] | Belgium | FRS-FNRS | 2011-15 | Archive | Application | All eligible | Small research (RC); research (RP) | X | X | X |  |  |
| Biernat, 2020 [28] | US | NIH | 2015 | Archive | Person | K awardees | K |  | X |  |  |  |
| Boyington, 2016  [29] | US | NHLBI | 2010-12 | Archive | Person | Early career PIs  w/unfunded R01s | R01 |  |  |  |  | X |
| Boyle, 2015 [30] | EU, UK | ERC, ERSC | 2007-13 | Archive | Application | All eligible | ERSC | X | X | X |  |  |
| Burns, 2019 [31] | Canada | CIHR | 2000-15 | Archive | Application | All eligible | Open operating grant program; new PI | X |  |  |  |  |
| Danish Indep Res Fund [32] | Denmark | DFF | 2020 | Archive | Application | All eligible | All | X | X | X |  |  |
| Dorismond, 2020 [33] | US | Triological Soc. Fdn, NIH | 2004-19 | Archive | Person | Fdn early career otolaryn-gologist  awardees | NIH grants (K, R01, other) |  | X |  |  | X |
| Dubosh, 2019 [34] | US | Gov, industry, intramural | 2012-18 | Archive | Application | Emergency med depts | Govt, Institutional, NIH, Industry, Organizational |  | X |  |  |  |
| Eloy, 2014 [35] | US | AAO-HNSF Fdn | 1985-2013 | Archive | Person | Early career CORE  awardees | CORE |  | X |  |  |  |
| Escobar-Alvarez, 2013 [36] | US | Doris Duke  Charitable Fdn | 2006-11 | Archive | Person | Early career  investigators | Career | X | X | X |  |  |
| Escobar-Alvarez,  2019 [37] | US | Doris Duke  Charitable Fdn | 2013-16 | Archive | Person | Early career  investigators | Career | X | X | X |  |  |
| Fabila-Castillo, 2019 [38] | Mexico | Mexican National  Council of Science | 2009-15 | Archive | Application | Basic science | Young Researcher;  Researcher | X | X | X |  |  |
| Fischer, 2010 [39] | Austria | FWF | 1999-2009 | Archive | Application | STEM, arts, humanities | Stand-alone  projects | X | X | X |  |  |
| Gallo, 2018 [40] | US | Unspecified | 2013-16 | Survey | Person | All eligible | Grant submission  or award, last 3 years | X | X | X |  |  |
| Gordon, 2009 [41] | US | Intramural | 2003-08 | Archive | Application | Pediatric residents | Intramural | X | X | X | X |  |
| Head, 2013 [42] | UK | Gov & fdn; UK & internat | 1997-2010 | Archive | Application | Infectious dz | Research & operations |  | X |  | X |  |
| Hechtman, 2018 [43] | US | NIH | 1991-2010,  follow-up to 2015 | Archive | Person | All eligible | RPGs |  |  |  |  | X |
| Heggeness, 2016  [44] | US | NIH | 2008-12 | Archive | Person | All eligible | K, RPG, R01 |  | X |  |  |  |
| Hosek, 2005 [45] | US | NSF, NIH, USDA | 2001-03 | Archive | Person | All eligible | Multiple | X (NSF, USDA  only) | X (USDA) | X (NSF, USDA) |  |  |
| Johnson et al., 2020 [46] | US | Hubble telescope | 2004-20 | Archive | Application | Astronomy | Telescope time | X | X | X |  |  |
| Kalyani, 2015 [47] | US | NIH | 1999-2008 | Archive | Person | JHU DOM faculty w/Ks | Research grants |  |  |  |  | X |
| Ledin, 2007 [48] | EU | EMBO | 2002-14 | Archive | Person | All eligible | Long-term  fellowships | X | X | X |  |  |
| Lee, 2005  [49] | US | NSF, DoE | 2000-01 | Archive | Person | Research center  affiliated faculty | "Research grant" |  |  |  | X |  |
| Ley, 2008 [50] | US | NIH | 2003-07 | Archive | Person | New or exper PI | K01, K08, K23, R01 | X |  | X |  |  |
| Materia, 2015  [51] | Italy | Emiglia-Romana  region | 2001-06 | Archive | Application | Agricultural science | Research grant | X |  |  |  |  |
| NIH [52] | US | NIH | 2005-20 | Archive | Application | All eligible | RPGs | X | X | X | X | X |
| NWO [53] | Nether-lands | NWO | 2005-10,  2014-18 | Archive | Application | All eligible | Talent; free comp,  soc chall | X | X | X |  |  |
| Okeigme, 2017 [54] | US | NIH | 1988-2009 | Archive | Person | Ob-Gyn K08, K12, K23 awardees | R, U, P |  |  |  |  | X |
| Pagel, 2015 [55] | US | Fdn for Anesthesia Education &  Research | 1987-2014 | Archive | Person | Anesthesia early career investigators | Small grants |  | X |  |  |  |
| Pohlhaus, 2011 [56] | US | NIH | 2008 | Archive | Both; Person data were used in analysis | All eligible | F, K, RP, R, T, U |  |  |  |  | X |
| Price, 2020 [57] | US | Dermatol.  Fdn | 2010-19 | Archive | Application | Dermatology | Career; research |  | X |  |  |  |
| Ripley, 2012 [58] | US | NIH | 2007 | Survey | Person | Mentored K awardees | Ks |  | X |  |  |  |
| Rissler, 2020 [59] | US | NSF | 2016 | Archive | Application | All eligible | All | X |  | X |  |  |
| Ross, 2016 [60] | US | NIH | 1978-2013 | Archive | Person | T32 trainees | F32s or Ks |  |  |  |  | X |
| Sandström, 2008 [61] | Sweden | Swedish Research Council Medicine Subcommittee | 2004 | Archive | Application | All eligible | Research projects | X | X | X |  |  |
| Sege, 2015 [62] | US | 2 Fdns | 2012-14 | Archive | Person | Early career  investigators | Research projects | X |  |  |  |  |
| Steinþórsdóttir,  2020 [63] | Iceland | IRF (gov); UIRF  (intramural) | 2013; 2010-  14 | Archive | Application | STEM, humanities,  education | Research projects | X (UIRF) | X | X(UIRF) | X |  |
| Sugimoto, 2017 [64] | US | NSF | 1981-2016 | Archive | Application | All eligible | All |  | X |  |  |  |
| Swaminathan, 2020 [65] | US | Intramural | 2010-17 | Archive | Person | Brigham Research Institute | Research; bridge; catalyst; interdiscip; micro; innovation | X | X | X |  |  |
| Swiss NSF [66] | Switzerland | Swiss NSF | 2005-20 | Archive | Application | All eligible | Projects; career;  programs | X | X | X | X |  |
| Tamblyn, 2016 [67] | Canada | CIHR | 2001-11 | Archive | Person | All eligible | Open |  | X | X |  | X |
| Tamblyn, 2018 [68] | Canada | CIHR | 2012-14 | Archive | Application | All eligible | Open; operating | X | X | X | X | X |
| Titone, 2018 [69] | Canada | NSERC | 2001-16  chair; 2009-16  all | Archive | Application | Cognitive science | Chair; discovery; postdoc |  | X |  | X |  |
| van den Besselaar, 2009 [70] | Netherlands | MAGW NWO | 2003-05 | Archive | Application | Social sciences | Open; Veni, Vidi,  Vici (Career) |  | X |  |  |  |
| van der Lee, 2015  [71] | Nether-lands | NWO | 2010-12 | Archive | Person | Early career  investigators | Veni (Career) |  |  |  |  | X |
| Wagner, 2007  [72] | US | Unspecified | 2004 | Survey | Person | Rehab med  professionals | "research grant" | X |  |  |  |  |
| Waisbren, 2008  [73] | US | Gov, fdn, industry | 2001-03 | Archive | Person | Harvard SOM faculty | Any | X | X | X |  | X |
| Weber-Main, 2020 [74] | US | Federal, non- federal (fdn, intramural) | 2015-19 | Archive | Person | Early career investigators in grant- writing coaching  programs | Any | X | X | X |  |  |
| Witteman, 2019 [75] | Canada | CIHR | 2011-16 | Archive | Person | All eligible | Foundation (scientist focus);  project | X | X | X |  |  |
| Yip, 2020 [76] | Hong Kong | Federal | 2015-21 | Archive | Person | UHK social science  faculty | Early career;  general research | X | X | X |  |  |
| Zhou, 2018 [77] | UK | Public,  philanthropic | 2000-13 | Archive | Application | Cancer research | Any |  | X |  | X |  |

^a^X=data available on this variable from this study.

Notes: AAO-HNSF=American Academy of Otolaryngology-Head and Neck Surgery Foundation; CDA=career development award; CIHR=Canadian Institutes of Health Research; CORE=Centralized Otolaryngology Research Efforts; DFF=Independent Research Fund Denmark; DoE=Department of Energy; EMBO=European Molecular Biology Organization; ERC=European Research Council; ERSC=UK Economic and Social Research Council; FRS-FNRS=National Fund for Scientific Research; IRF=Icelandic Research Fund; JHU=Johns Hopkins University; MAGW=Netherlands Economic and Social Science Research Council; NHLBI=National Heart, Lung and Blood Institute; NHMRC=National Health and Medical Research Council; NIH=National Institutes of Health; NSERC=National Sciences and Engineering Research Council; NSF=National Science Foundation; NWO=Netherlands Research Council; SOM=School of Medicine; UHK=University of Hong Kong; UIRF=University of Iceland Research Fund; USDA=United States Department of Agriculture.

eTable 2. Included Studies’ Gender Bias Conclusion and Comparisons to Eligible Proportions of Women

| **1^st^ author, year [ref #]** | **% Women outcomes (see eFigures 1, 2, 13, 14)** | **Application outcomes comparisons** | **Award outcomes comparisons** | **% Women eligible (see eFigure 22) and source^1^** | **Gender bias?** |
| --- | --- | --- | --- | --- | --- |
| Akabas, 2019 [23] | Awards = 20% |  | < | 27%, p. 3, Figure 1 totals | Y (“fewer women have NIH grants”) |
| Andersson, 2021 [24] | Apps = 43%; awards = 37% | < | < | 53%, KI only p. 67^2^ | Y (“Accumulating gender bias is quantifiable”) |
| Australian Gov, 2019-20 [25] | Apps = 47%; awards = 42% | > | = | 42%^3^ | Y (“Proportion of female applicants fell progressively with seniority…research led by women (is) an area of need”) |
| Bautista-Puig, 2019 [26] | Apps = 25%, awards = 22% | < | < | 41% EU-28, Annex 4.2 for 2012^4^ | Y (“grants are not equitably distributed”) |
| Beck, 2017 [27] | Apps = 30%; Awards = 27% | > | = | 27%, p. 122 | N (“no significant influence of gender”) |
| Biernat, 2020 [28] | K awards = 50% |  | > | 44%, asst profs^5^ | Y (“gender bias in grant reviews”’) |
| Boyington, 2016  [29] | Re-apps of unfunded = 32% | < |  | 37%, Table 3 | N/A |
| Boyle, 2015 [30] | Apps = 41%; awards = 41% | < | < | 45%, ay14-15 social studies cost center^7^ | N (“UK social science funding does not show such gender bias”) |
| Burns, 2019 [31] | Apps = 32% | = |  | 33%, Figure 1^8^ | Y (“Gender disparity existed overall in grant and personnel award success rates…”) |
| Danish Indep Res Fund [32] | Apps = 33% | < |  | 36%, DK, Annex 4.1^9^ | N/A |
| Dorismond, 2020 [33] | Awards= 33%, NIH apps after fdn award = 29% | > | > | 17%, Otolaryngology^10^ | Y (“female recipients lag behind their male colleagues”) |
| Dubosh, 2019 [34] | Awards = 27% |  | < | 31%, p. 1358, table 1 | Y (“gender discrepancies exist among funded grants of EM faculty” |
| Eloy, 2014 [35] | Awards = 26% |  | < | 29%, Table 3, Otolaryngology^6^ | N/A |
| Escobar-Alvarez, 2013 [36] | Apps = 40%, awards = 34% | < | < | 43%, Table 3^6^ | N (“female applicants… were as successful as the male applicants” |
| Escobar-Alvarez,  2019 [37] | Apps = 43% ; awards = 24% | = | < | 44%, asst profs^5^ | N (“being female had… no significant association (with award attainment after accounting for having a PhD and research effort) |
| Fabila-Castillo, 2019 [38] | Apps = 34%; Awards = 32% | = | = | 32%^11^ | +/- (“possible indirect gender bias ») |
| Fischer, 2010 [39] | Apps = 18%, awards = 17% | < | < | 27%, AT, Annex 1.1^12^ | N (“No evidence… that women placed at a disadvantage in the peer review process…(lower approval rate probably linked) to the underrepresentation of women… family-related burdens… unstable employment.” |
| Gallo, 2018 [40] | Apps = 35%; awards = 29% | = | < | 33%, p. 219 Table 1 survey respondents | N/A |
| Gordon, 2009 [41] | Apps = 61%; awards = 55% | < | < | 67%, p. e357 "two-thirds of all (327) residents were female" | Y (“Gender differences existed in research grant applications and funding.”) |
| Head, 2013 [42] | Awards = 28% |  | < | 37%, UK, Annex 1.1^12^ | Y (“cannot comment on the extent of inequalities or bias…our analysis shows an unequal distribution of investments in infectious disease research for men and women.”) |
| Hechtman, 2018 [43] | Reapp = 30% | > |  | 26%, p. 15, Table 1, new Type 1 applications [56] | N (“men’s and women’s generally comparable funding longevities…”) |
| Heggeness, 2016  [44] | Awards = 47% |  | < | 52%, Supplemental digital content number of postdocs | +/- (“women are overrepresented in training and early career programs and underrepresented in independent research awards”) |
| Hosek, 2005 [45] | NSF apps = 21%; USDA apps = 23%; USDA awards = 23% | < NSF;  < USDA | < USDA | 26%, Data S1, 2001-2003, all ranks [59] | +/- (“With two important exceptions (award amounts; likelihood to re-apply), we did not find gender differences in federal grant funding outcomes”) |
| Johnson et al., 2020 [46] | Apps = 23%; awards = 20% | < | < | 27%, 2016 new hires^13^ | Y (“a statistical bias existed…”) |
| Kalyani, 2015 [47] | R-award after K = 50% |  | > | 45%, p. 937 | N (“No sex differences were found…” |
| Ledin, 2007 [48] | Apps = 45%; awards = 41% | > | > | 33%, EU, Annex 1.1, 2006^14^ | Y (“subtle differences… combine to real effects that are reflected in lower success rates for women” |
| Lee, 2005  [49] | N/A amount data only |  |  |  | N/A |
| Ley, 2008 [50] | K08 apps = 31%; K23 apps = 51%; K01 apps = 52% | < K08  > K23, K01 |  | 37%, Table 3, 1997 cohort of Asst Profs^15^ | N (“Women are equally… successful at obtaining NIH grants…”) |
| Materia, 2015  [51] | Apps = 13% | = |  | 13%, IT, Annex 3.2 Grade A, agricultural sciences, 2007^15^ | Y (“male reviewers are more likely to be selected (and) receive funds”) |
| NIH [52] | Apps = 31%; awards = 30%; re-apps = %; comp renewals = 30% | < | < | 41%, Totals 2013-2020^16^ | N/A |
| NWO [53] | Talent apps = 29%; talent awards = 40%; social chall apps = 26%;; social chall awards = 25%; free comp apps = 19%; free comp awards = 19% | > talent;  = social chall apps;  < free comp apps | > talent awards;  = social chall awards;  < free comp awards | 24%, Annex 1.1^4^, Annex 4.1^9,12,14,17^ | +/- (Some years had no gender comparisons in the report, others did.) |
| Okeigwe, 2017 [54] | Reapps = 62%; re-awards = 60% (K to indep funding) | > | > | 54%, Table 3, Ob/gyn faculty 2012^6^ | N (“Our analysis shows that sex… did not impact… ability to obtain independent funding” |
| Pagel, 2015 [55] | Awards = 21% |  | < | 35%, Anesthesiology faculty, Table 3^5^ | N (“there were no gender-based differences in NIH funding”) |
| Pohlhaus, 2011 [56] | K to R01 apps = 36%; R03/21 to R01 apps 37%; K to R01 awards = 35%; R03/21 to R01 awards 30% | < | < | 47%, p. 14, Chart 1, early mentored K applicants | +/- (“success rates (except for subsequent grants)… were not significantly different in most award programs”) |
| Price, 2020 [57] | Awards = 48% |  | = | 49%, Dermatology faculty, Table 3^5^ | N (“has a more equal sex distribution among recipients”) |
| Ripley, 2012 [58] | Awards = 52% |  | > | 47%, supplemental content on K awardees [44] | N (“the gender of K recipients was evenly divided”) |
| Rissler, 2020 [59] | Apps = 27% | < |  | 32%, Data S1, 2008-2015, all ranks | +/- (“women are as likely to be funded… but the percentage of women submitting proposals was less than expected…”) |
| Ross, 2016 [60] | Awards after T32 = 60% |  | < | 67%, per email | N/A |
| Sandström, 2008 [61] | Apps = 36%; awards = 29% | < | < | 48%, SE, Annex 1.2, 2005^14^ | Y (“Nepotism seems to be a persistent problem in the Swedish peer review system”) |
| Sege, 2015 [62] | Apps = 42% | = |  | 43%, Asst Profs in Table 3^6^ | Y (“Women received significantly less start-up support from their institutions than men”) |
| Steinþórsdóttir,  2020 [63] | UIRF apps = 40%;  UIRF awards = 39%;  IRF awards = 33%; | = | = UIRF,  < IRF | 39%, p. 368, Table 1 | Y (“the funding system is biased in favour of men…”) |
| Sugimoto, 2017 [64] | Awards = 19% |  | < | 32%, Data S1, 2008-2015, all ranks [59] | Y (“(there is) progress towards parity… and there is still work to be done”) |
| Swaminathan, 2020 [65] | Apps = 35%; awards = 33% | < | < | 39%, p. 5, Table 2, US medical school faculty | N (“We did not detect a significant bias by gender…”) |
| Swiss NSF [66] | Project apps = 22%; career apps = 42%; program apps = 22%; project awards = 19%; career awards = 42%; program awards = 21% | < project & programs  > career | < project & programs  > career | 33%, CH, Annex 4.1^4,17^ | N/A |
| Tamblyn, 2016 [67] | Awards = 28%; re-apps = 29%; re-awards = 24% | < | < | 43%, Table 1, 2011, life sciences^18^ | Y (“male researchers were more likely to be funded than female researchers”) |
| Tamblyn, 2018 [68] | Apps = 34%; awards = 32%; re-apps = 32% | < | < | 43%, Table 1, 2011, life sciences^18^ | Y (“There is evidence of bias in peer review…”) |
| Titone, 2018 [69] | Chair awards = 45%; Discovery awards = 36% |  | = Chair; < discovery | 43%, Table 1, 2011, life sciences^18^ | Y (“gender parity issues remain a concern…”) |
| van den Besselaar, 2009 [70] | Awards = 29% |  | < | 35%, Asst Profs, 2003-2005, beh & soc sci, econ, law^19^ | +/- (“Male researchers receive higher scores from referees… final decision-making process corrects the results in favor of female applicants…”) |
| van der Lee, 2015  [71] | Reapp of unfunded apps = 45%; re-app awards = 45% | = | = | 43%, p. 12350, Ns eligible to re-apply (N applications - N awards) | Y (“ …compelling evidence of gender bias…”) |
| Wagner, 2007  [72] | Apps = 33% | < |  | 39%, p. 561, survey respondents involved in research | Y (“women… are less successful than their male counterparts”) |
| Waisbren, 2008  [73] | Apps = 26%; awards = 24%; reapps = 23%; re-app awards = 21% | < | < | 35%, p. 212, Table 2 | N (“Gender disparity in grant funding is largely explained by academic rank”) |
| Weber-Main, 2020 [74] | Apps = 70%; Awards = 72% | > | > | 67%, p. 16, Table 5 | N/A – reducing disparities assumes they exist |
| Witteman, 2019 [75] | Apps = 34%; awards = 31% | < | < | 43%, Table 1, 2011, life sciences^18^ | Y (“gender gaps in grant funding are attributable to less favourable assessments of women as investigators”) |
| Yip, 2020 [76] | Apps = 43%; awards = 46% | = | > | 43%, p. 5, Table 1 | N (“lack of gender bias”) |
| Zhou, 2018 [77] | Awards = 31% |  | < | 38%, Annex 1.1^12^, Annex 4.1^4^ | Y (“substantial differences in cancer research investment by gender”) |

Note: AT = Austria; AU = Australia; CA = Canada; CH = Switzerland; EM = emergency medicine; IT = Italy; N/A = not applicable: these studies included gender data but did not comment on it; NL = Netherlands; PMR = physical medicine and rehabilitation; SE = Sweden; US = United States

^1^Superscripts denote website used

^2^https://english.uka.se/download/18.7f89790216483fb85588e86/1534509947612/Report-2018-06-26-higher-education-in-Sweden-2018.pdf

^3^https://www.education.gov.au/higher-education-statistics/resources/2020-staff-fulltime-equivalence

^4^She Figures 2015 https://op.europa.eu/en/publication-detail/-/publication/f546dfed-41a9-11e6-af30-01aa75ed71a1#

^5^https://www.aamc.org/media/8671/download

^6^https://www.hopkinsmedicine.org/women_science_medicine/_pdfs/women_in_u_s_academic_medicine_statistics_and_benchmarking_report_20112012.pdf

^7^<https://www.hesa.ac.uk/data-and-analysis/staff/chart-5>

^8^https://www.universityaffairs.ca/features/feature-article/history-canadas-full-time-faculty-six-charts/

^9^She Figures 2021 https://ec.europa.eu/assets/rtd/shefigures2021/index.html

^10^https://www.aamc.org/data-reports/workforce/interactive-data/active-physicians-sex-and-specialty-2017

^11^32% W researchers in MX (<https://uis.unesco.org/sites/default/files/documents/fs34-women-in-science-2015-en.pdf>) with 281.32424 researchers/1M pop and 1233.333M pop (http://data.uis.unesco.org/#)

^12^She Figures 2012 https://op.europa.eu/en/publication-detail/-/publication/ba8dc59b-61b8-4c03-9176-373fd9ddac82

^13^https://www.aip.org/statistics/reports/women-physics-and-astronomy-2019

^14^She Figures 2009 https://op.europa.eu/en/publication-detail/-/publication/6358e1d9-385c-4961-946e-52ed66de5bbb/language-en

^15^https://www.aamc.org/media/41776/download

^16^<https://www.aamc.org/data-reports/faculty-institutions/interactive-data/us-medical-school-faculty-trends-counts>

^17^She Figures 2018 https://research-and-innovation.ec.europa.eu/knowledge-publications-tools-and-data/publications/all-publications/she-figures-2018_en

^18^https://www150.statcan.gc.ca/n1/pub/75-006-x/2016001/article/14643-eng.htm

^19^https://www.universiteitenvannederland.nl/en_GB/f_c_ontwikkeling_aandeel_vrouwen.html

**eResults**

eFigure 1. Gender Differences in Award Acceptance Rates in U.S. versus non-U.S. Studies: Forest Plot

eFigure 2. Gender Differences in Award Acceptance Rates: Doi Plot


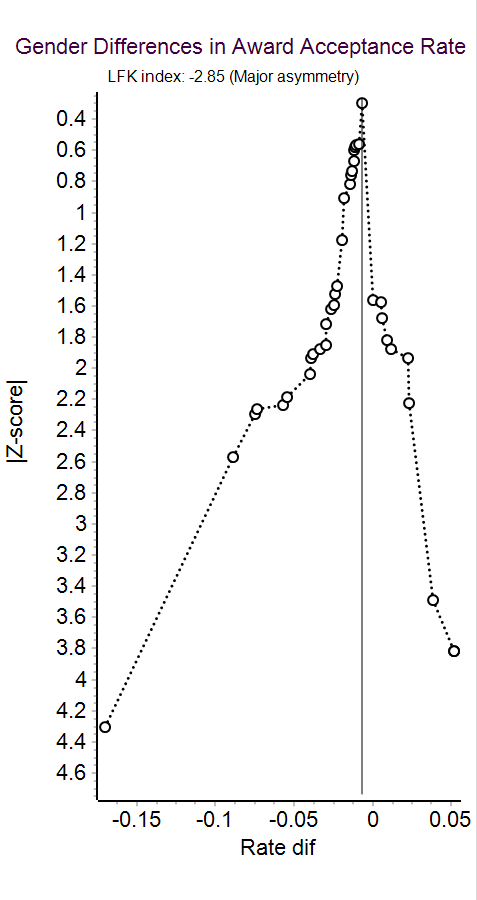


eFigure 3. Gender Differences in Award Acceptance Rates: Sensitivity Analysis


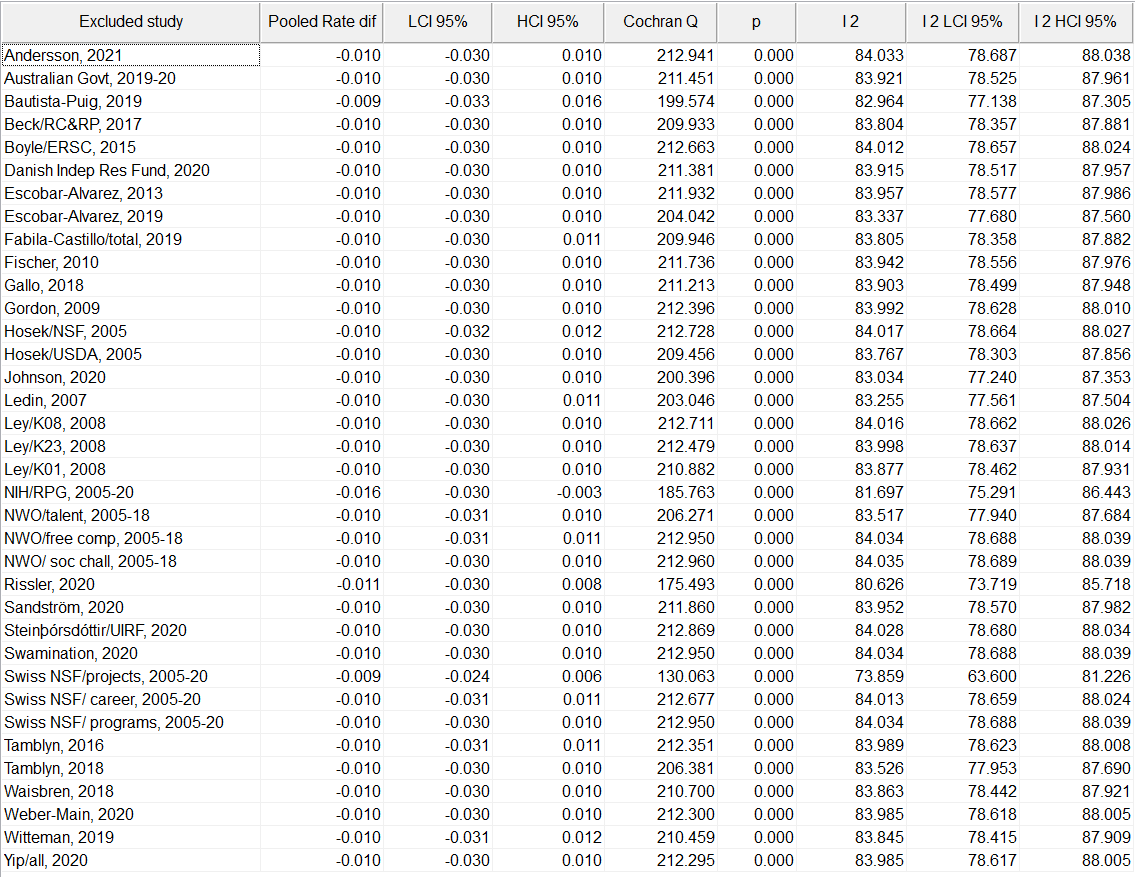


eFigure 4. Gender Differences in Reapplication Award Acceptance Rates (Awards after Reapplication among Previous Applicants and Awardees): Forest Plot

eFigure 5. Gender Differences in Reapplication Award Acceptance Rates (Awards after Reapplication among Previous Applicants and Awardees): Doi Plot

eFigure 6. Gender Differences in Reapplication Award Acceptance Rates (Awards after Reapplication among Previous Applicants and Awardees): Sensitivity Analysis


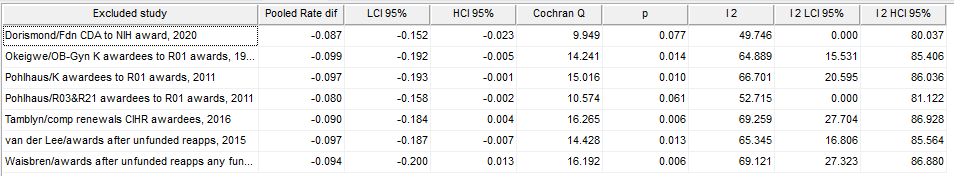


eFigure 7. Gender Differences in Award Amounts in U.S. versus non-U.S. Studies: Forest Plot

eFigure 8. Gender Differences in Award Amounts: Doi Plot

eFigure 9. Gender Differences in Award Amounts: Sensitivity Analysis


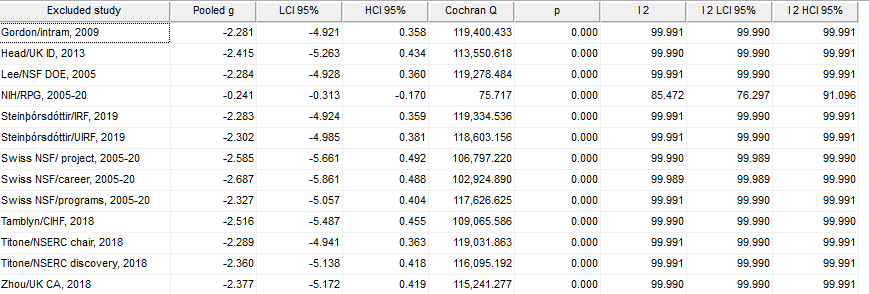


eFigure 10. Gender Differences in the Proportions of Applications in U.S. versus non-U.S. Studies: Forest Plot

eFigure 11. Gender Differences in the Proportions of Applications: Doi Plot


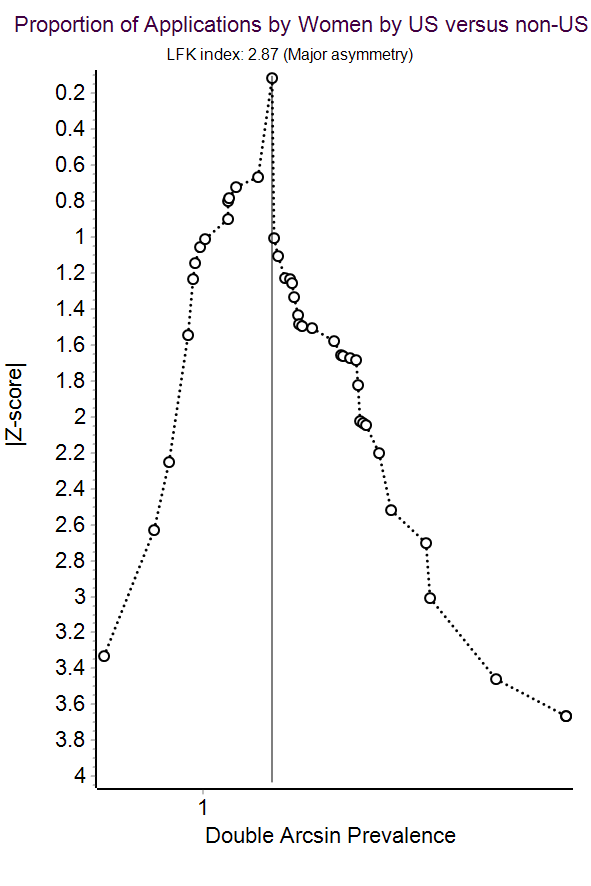


eFigure 12. Gender Differences in the Proportions of Applications: Sensitivity Analysis


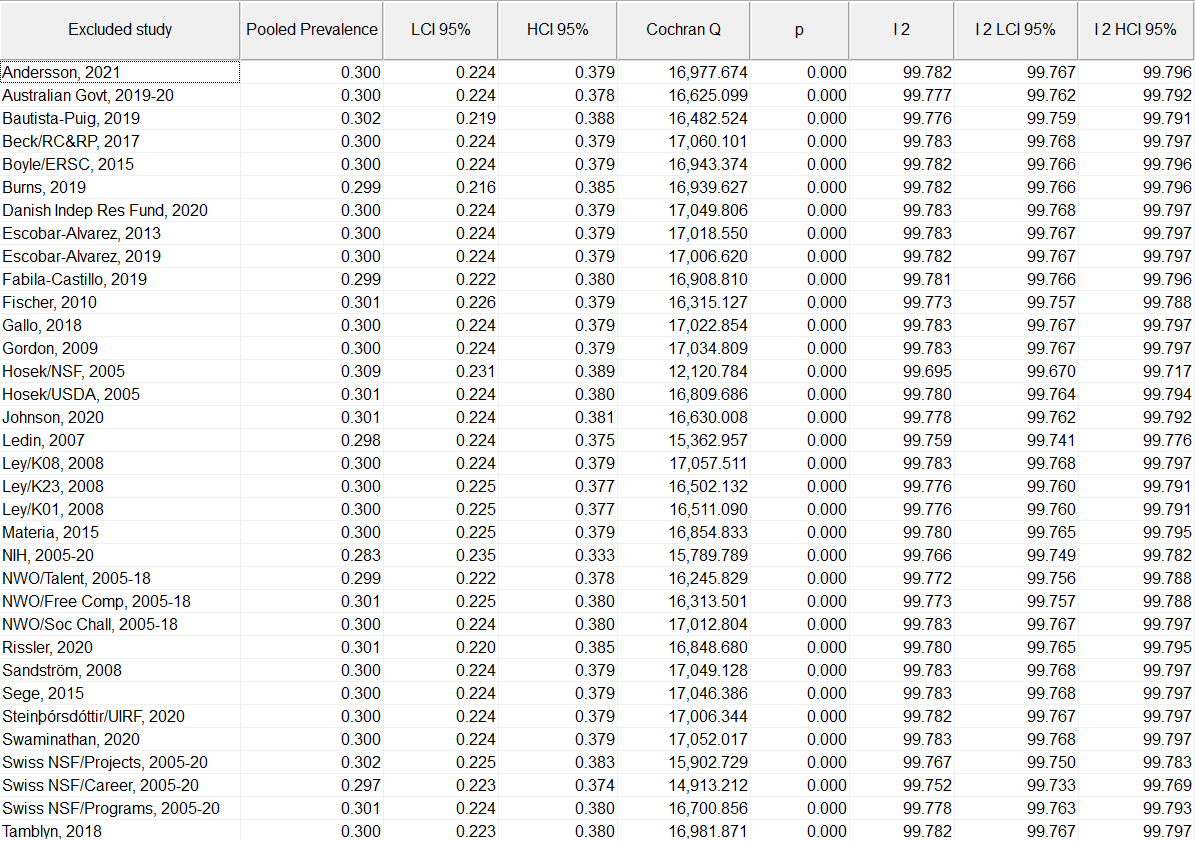


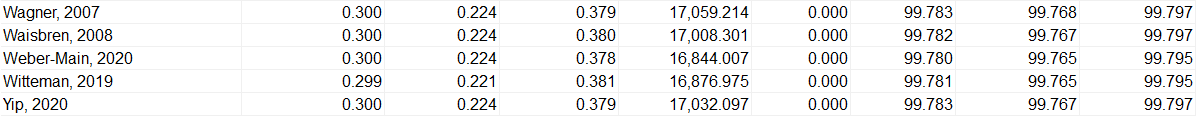


eFigure 13. Gender Differences in the Proportions of Reapplications: Forest Plot

eFigure 14. Gender Differences in the Proportions of Reapplications: Doi Plot

eFigure 15. Gender Differences in the Proportions of Reapplications: Sensitivity Analysis

**
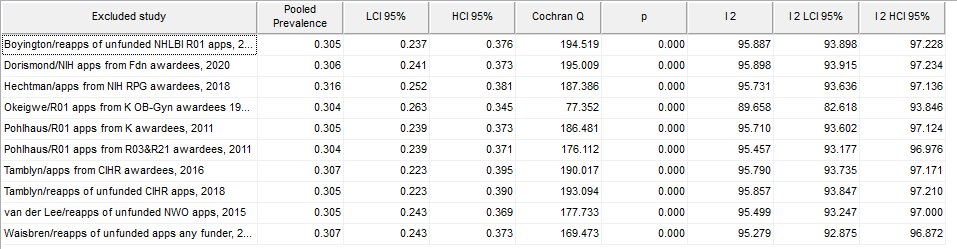
**

eFigure 16. Gender Differences in the Proportions of Awards in U.S. versus non-U.S. Studies: Forest Plot

eFigure 17. Gender Differences in the Proportions of Awards: Doi Plot


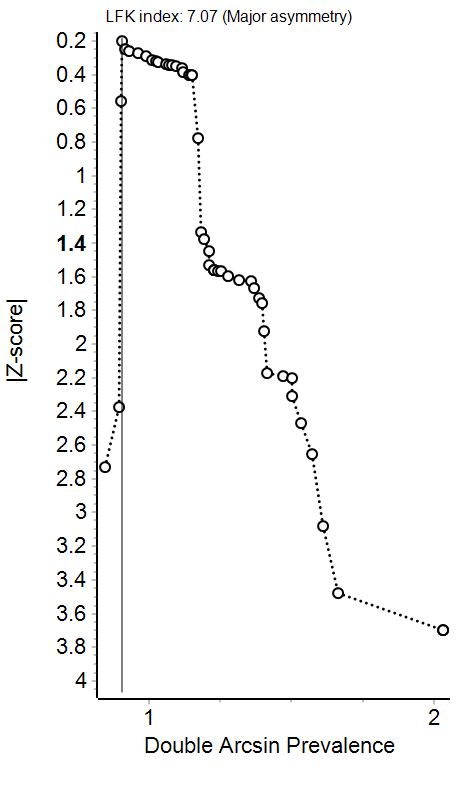


eFigure 18. Gender Differences in the Proportions of Awards: Sensitivity Analysis

**
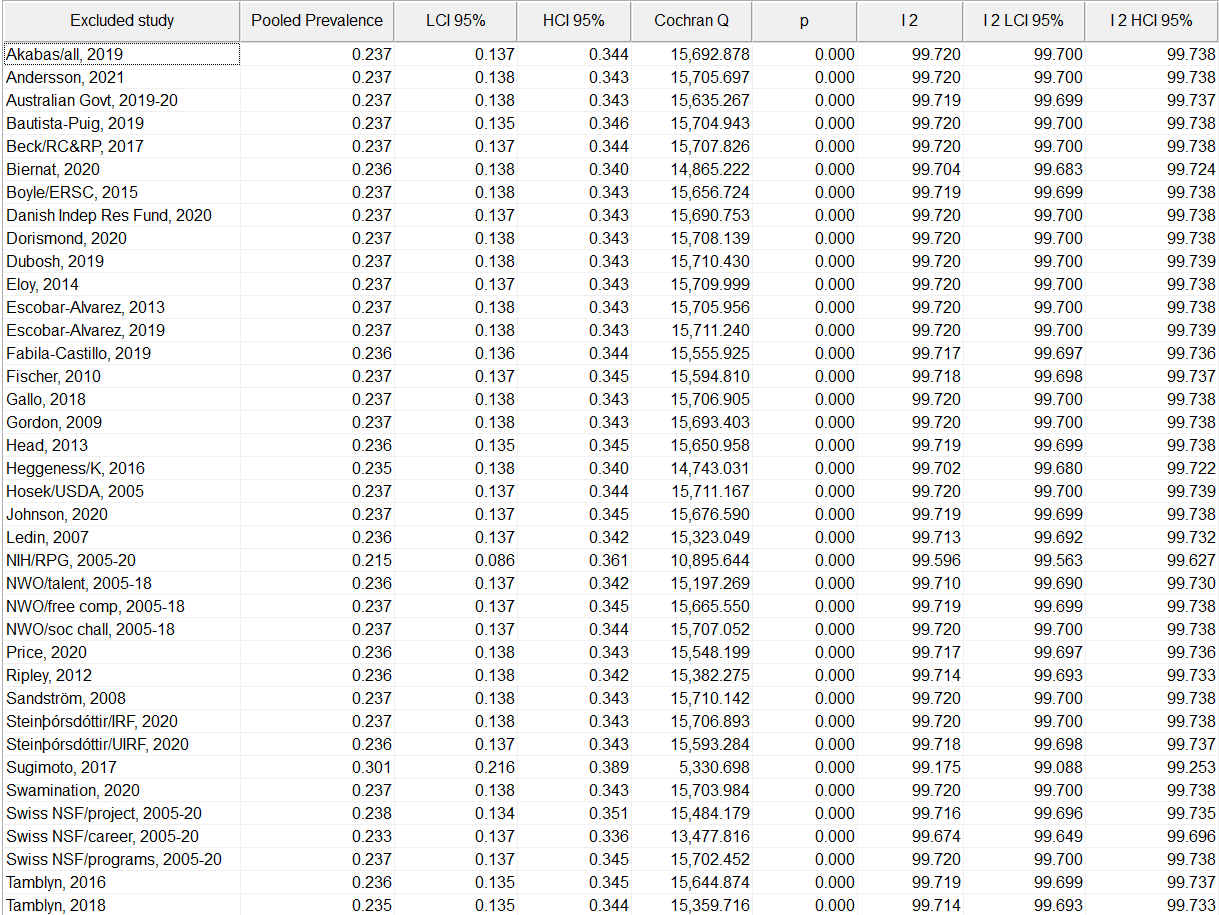
**
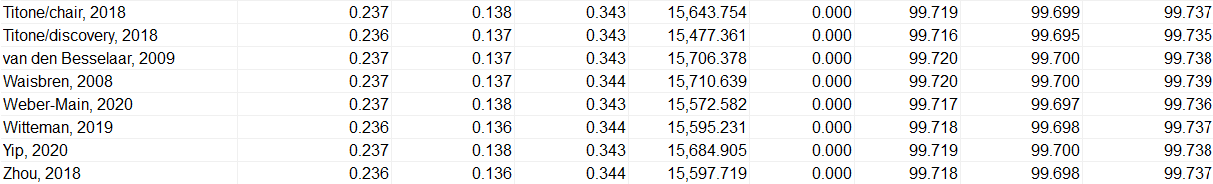


eFigure 19. Gender Differences in Proportions of Awards after Reapplication: Forest Plot

eFigure 20. Gender Differences in Proportions of Awards after Reapplications: Doi Plot

eFigure 21. Gender Differences in Proportions of Awards after Reapplications: Sensitivity Analysis

**
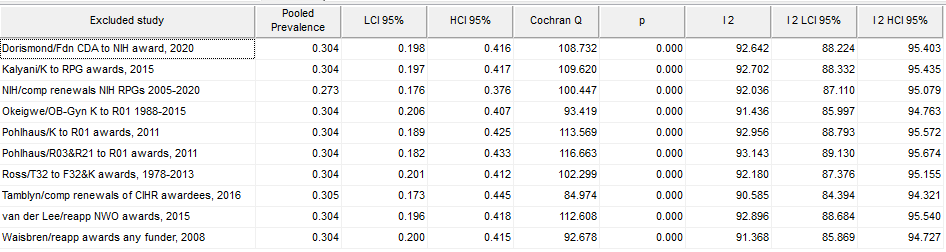
**

eFigure 22. Proportion of Eligible Women Applicants: Forest Plot

eFigure 22. Proportion of Eligible Women Applicants: Doi Plot


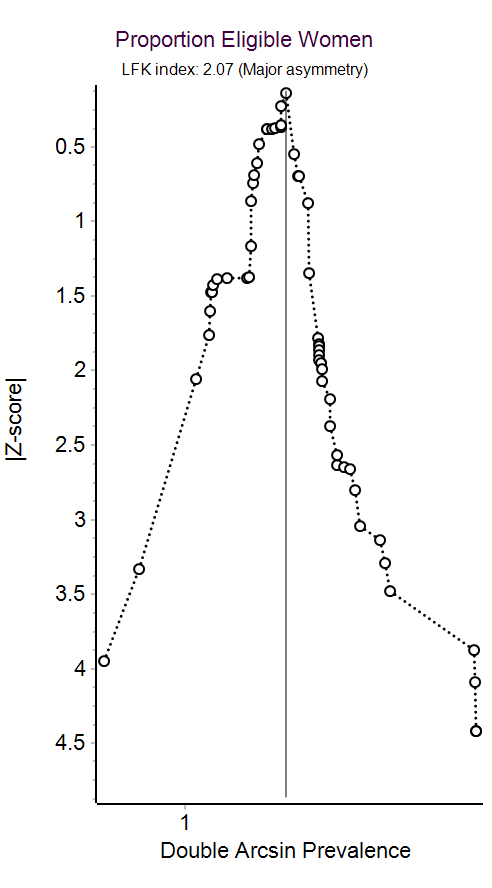


eFigure 22. Proportion of Eligible Women Applicants: Sensitivity Analysis


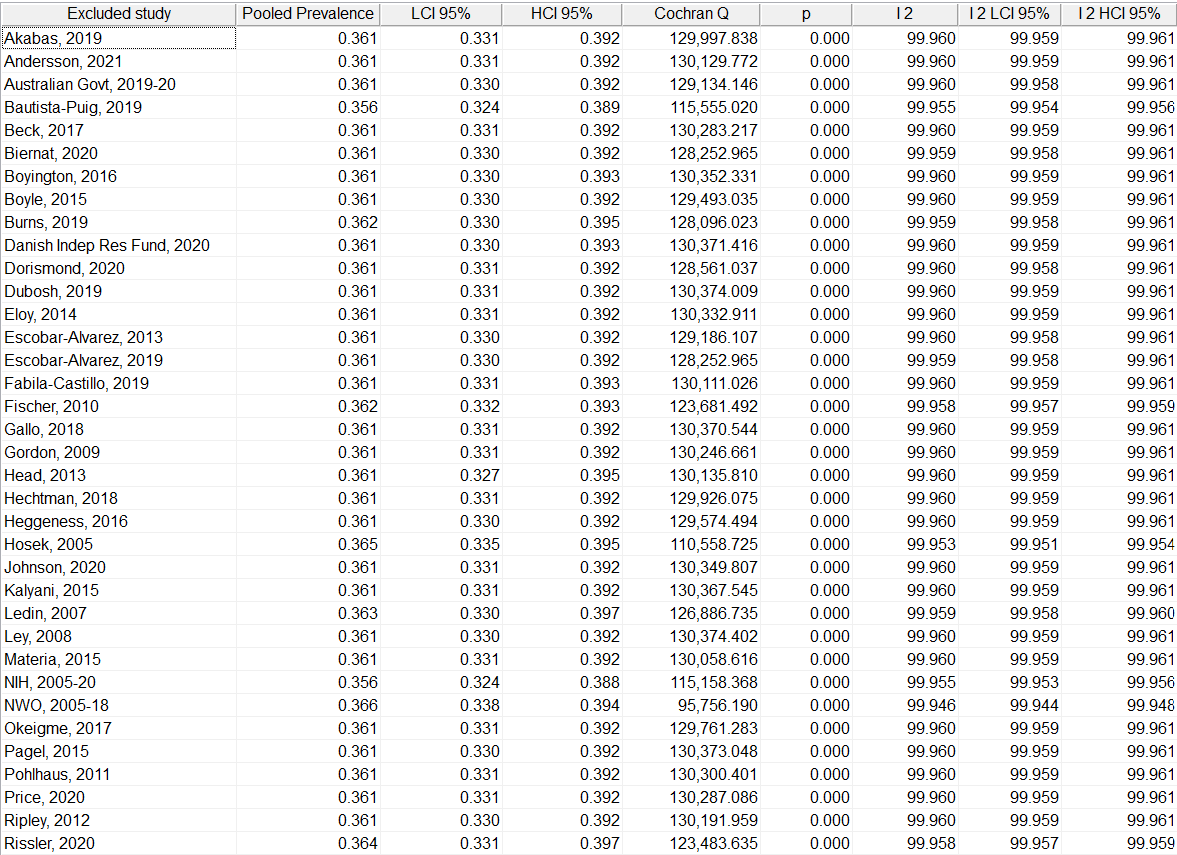


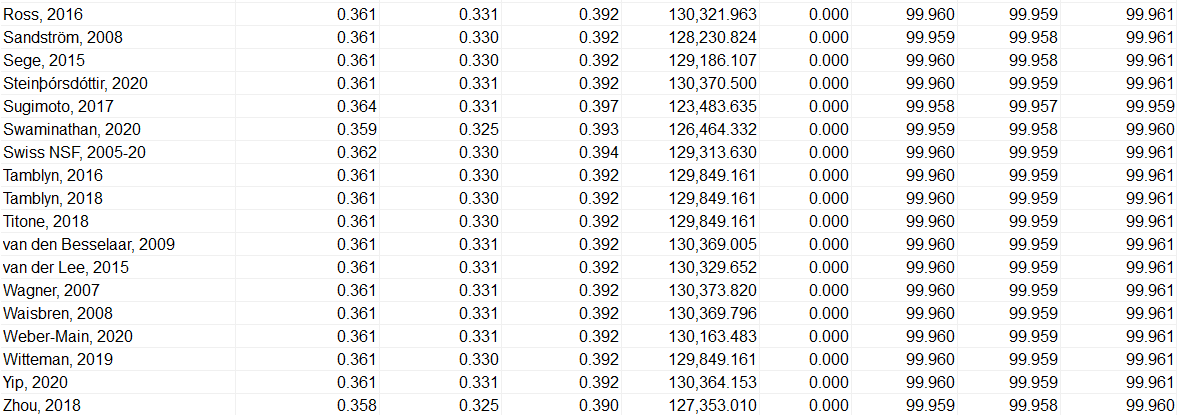


**eDiscussion**

**Search to estimate the number of funders**

Pivot-RP (ExLibris, a ProQuest company, 2022) was used to estimate the number of funders in the sciences in the geographical areas represented by the studies in the study and yielded 8,857 results. The search syntax follows:

**Search:**(sponsor_type:(academic OR us_government OR non_us_government OR public_sector_local OR multinational OR commercial OR society OR foundation) AND (keyword:(Agriculture and Food Sciences OR Energy Sciences OR Engineering OR Health and Medicine OR Natural and Physical Sciences, Mathematics and Technology OR Social Sciences) OR keyword_parent:(Agriculture and Food Sciences OR Energy Sciences OR Engineering OR Health and Medicine OR Natural and Physical Sciences, Mathematics and Technology OR Social Sciences)) AND applicant_type:(indiv_early_career OR indiv_mid_career) AND (funder_location:(Australia OR Austria OR Belgium OR Canada OR Denmark OR European Commission OR Hong Kong OR Iceland OR Italy OR Mexico OR Netherlands OR Sweden OR Switzerland OR United Kingdom OR United States)) AND award_type:(trial OR research OR fellowship OR equipment OR curriculum))

**Re-analysis of Bornmann et al., 2007 data**

Twenty-two sources provided data on the proportion of applications and awards. Regarding applications, women accounted for 21% of applications (95% CI 6% to 38%). There was significant heterogeneity in effect sizes (*Q* = 10837.80, *p* < .001, *I*^2^ = 100%): sources’ numbers of applications ranging from 114 to 254,438. The measure of reporting bias (LFK = 1.32) favored studies reporting higher application prevalence among women, suggesting that reports of lower prevalence may be underrepresented.

Regarding awards, women accounted for 19% of applications (95% CI 6% to 34%). There was significant heterogeneity in effect sizes (*Q* = 2992.29, *p* < .001, *I*^2^ = 99%): sources’ numbers of awards ranging from 20 to 78,509. There was no significant reporting bias (LFK = 0.95).

eFigure 22. Gender Differences in Applications in Bornmann et al. (2007) data: Forest Plot

eFigure 23. Gender Differences in Awards in Bornmann et al. (2007) data: Forest Plot
